# NPK nano-fertilizers enhance growth, oil quality, and yield regularity in Picual olive trees

**DOI:** 10.1038/s41598-025-17267-9

**Published:** 2025-09-12

**Authors:** Ibrahim Hmmam, Hassan Mahmoud Korkar, Islam Ahmed, Asmaa Gamal Abd El-hamied, Ayman Shaban

**Affiliations:** 1 Pomology Department, Faculty of Agriculture, Cairo University, PO box 12613, Giza, Egypt; 2 The Higher Institute for Agricultural Co-Operation, Cairo, Egypt; 3 Oils and Fats Research Department, Food Technology Research Institute, Agricultural Research Center, Giza, Egypt

**Keywords:** Biennial bearing, Flowering, Fruiting, NPK nano-fertilizers, Oil quality, Picual olive, Vegetative growth, Yield, Plant sciences, Plant physiology, Nanoparticles

## Abstract

This study aimed to assess the impact of green-synthesized NPK nano-fertilizers (NPKNF) on the growth, productivity, and quality of the ‘Picual’ olive cultivar under Egyptian conditions. Foliar sprays of NPKNF at 0 (control), 2000, 3000, and 4000 mg L^−1^ were applied during the “on-year” season. Evaluations covered vegetative growth, leaf and fruit mineral content, fruit traits, yield, oil and moisture content, and various olive oil quality indicators. In the following “off-year” season, flowering, fruit set, yield, and alternate bearing severity were also examined. Results indicated a significant enhancement in vegetative growth, fruit traits, yield, and oil properties during the “on-year” season in response to increasing NPKNF concentrations. The highest concentration (4000 mg L^−1^) demonstrated the most pronounced improvements across all evaluated parameters compared to the untreated control. Treated trees produced larger fruits with an increased pulp-to-pit ratio and a higher oil content. Oil extracted from NPKNF-treated fruits exhibited a measurable reduction in total phenolic compounds (289.28, 298.59, and 277.11 mg/kg with 2000, 3000, and 4000 mg L^−1^, respectively) compared to the untreated control (350.51 mg/kg). These compounds act as natural antioxidants and contribute to the nutritional value and organoleptic properties of olive oil. Despite this reduction, all olive oil samples were classified as extra virgin, with sensory analysis indicating a moderate fruitiness level across treatments. Furthermore, an improvement in olive flowering, fruiting parameters, and yield during the “off-year” was observed, along with a reduction in the severity of alternate bearing in response to NPKNF treatments. One of the most significant outcomes of the study, consistent with its objective, is the significant increase in the number of new shoots per shoot during the “on-year,” reaching 5.09 with NPKNF 4000 compared to 1.03 in the control. This increase contributes to the next season’s yield (“off-year”) by reducing the impact of alternate bearing, as evidenced by the significant difference in severity values, with 85.40 for NPKNF 4000 and 92.66 for the control. These findings emphasize the potential role of NPKNF treatments in enhancing olive fruit quality and oil properties, while also contributing to the mitigation of alternate bearing severity.

**Supplementary Information:**

The online version contains supplementary material available at https://doi.org/10.1038/s41598-025-17267-9.

## Introduction

The olive tree (*Olea europaea* L.), a member of the Oleaceae family, is an evergreen species recognized as one of the earliest cultivated trees in human history^1^. Notably, the Mediterranean region accounts for approximately 99% of global olive oil production and 87% of its consumption^2^. Olive trees thrive in arid, and semi-arid, and newly reclaimed lands due to their resilience to drought, temperature fluctuations, and salinity^3–5^. Their production is economically vital in Egypt and Mediterranean regions, serving pickling, oil extraction or both^3,4,6,7^. Olive oil and table olives, are rich sources of monounsaturated fatty acids and antioxidants, with notable nutritional and pharmacological benefits^4,8^. In 2022, global olive production reached 18.4 million tons from 11 million hectares, yielding 3.82 million tons of olive oil and 3.25 million tons of table olives^9^. Egypt, a key producer, has expanded cultivation to 280,000 faddans under its 2030 agricultural plan, producing 40,000 tons of olive oil and 600,000 tons of table olives annually, ranking first globally in table olive output^9,10^.

Olive production faces challenges from alternate bearing, a cyclical pattern of irregular yields^11,12^. In “on-years,” high fruit production diverts nutrients from vegetative growth, reducing subsequent yields (“off-years”)^13–15^. Conversely, “off-year” promote vegetative growth and flower bud formation, boosting the next season’s harvest^15^. Alternate bearing severity in olive trees is strongly influenced by nutrient availability, particularly NPK fertilization^12,16^. Balanced NPK fertilization contributes to the regulation of the annual growth cycle in olive trees. Specifically, nitrogen enhances chlorophyll concentration in leaves^17^ and stimulates photosynthetic activity^18^, promoting shoot elongation^19,20^ and flowering initiation^16^. Supplemental nitrogen application prior to flowering and fruit set improves reproductive performance^15^ while increasing the tree’s ability to assimilate other essential nutrients^21^. Phosphorus serves essential roles in fundamental plant processes including cell division, energy transfer, nucleic acid synthesis, carbohydrate metabolism, photosynthesis, respiration, and nitrogen fixation^22–25^. Potassium contributes significantly to physiological functions such as osmoregulation, carbohydrate translocation, membrane potential maintenance, and stress adaptation^26–28^. It also actively participates in stomatal regulation^26,29,30^ and supports photosynthetic efficiency^26,31^.

Conventional foliar fertilizers exhibit limited efficiency due to their large particle size (> 100 nm), which restricts leaf absorption^32^. Nano-fertilizers, with ultrafine particles (< 100 nm), offer a promising solution, improving uptake efficiency and serving as a sustainable alternative in modern agriculture^32–36^. These fertilizers demonstrate comparable or superior performance to conventional NPK in promoting crop growth and yield, even at reduced application rates. Research on fruit trees, including citrus^33^, date palm^34,35^, grapevines^36^, mango^37–39^, pomegranate^40^, wild pear seedlings^41^, and sapota^42^, confirms in some cases that nano NPK enhances plant growth, yield, and fruit quality, often at half the standard dose, without compromising productivity even when combined with reduced conventional fertilization. Similar results were observed in rice, capsicum, lupine, spinach, and cabbage using waste-derived nano NPK and nano-biofertilizers, which performed as well as or better than traditional fertilizers^43–48^.

This study establishes the first field-scale assessment of foliar-applied NPK nano-fertilizers (NPKNF) in mature Picual olive orchards under Egyptian arid conditions, significantly extending previous greenhouse-based seedling research^49,50^. Our work has three primary objectives: First, to quantify NPKNF effects on vegetative growth dynamics and reproductive development, including flowering patterns and yield parameters. Second, to evaluate the impact of NPKNF on olive oil quality characteristics. Third, to mitigate the alternate bearing through “on-year” NPKNF applications that regulate subsequent “off-year” yields. Through this multifaceted investigation, we bridge critical knowledge gaps between nanotechnology innovation and practical olive cultivation in arid ecosystems.

## Materials and methods

The present study was conducted over two consecutive seasons, 2022/2023 and 2023/2024, at the orchard of the Higher Institute for Agricultural Cooperation, located along the Cairo-Alexandria Desert Road in Egypt (30° 44′ 47″ E 30° 10′ 15″ N). The experiment utilized 20-year-old ‘Picual’ olive trees, planted at a spacing of 6  × 6 meters in sandy soil and irrigated using a drip irrigation system. The ‘Picual’ olive cultivar is vigorous, characterized by large, elongated fruits, high oil content, and rapid ripening. This dual-purpose cultivar is suitable for both pickling and oil extraction. It exhibits a short juvenile phase along with strong heat tolerance and moderate cold resistance. However, it shows moderate sensitivity to salt stress and susceptibility to Verticillium wilt.

The irrigation water had a salinity level of approximately 4400 mg L^−1^ (Table 1). The meteorological data for the experimental period, including maximum temperature (°C), minimum temperature (°C), average temperature (°C), relative humidity (%), and annual precipitation (mm), are provided in Supplementary Table S1.

**Table 1:** Chemical analysis of irrigation water.

pH	EC (ds m^− 1^)	TDS (mg L^−1^)	Cations (meq L^− 1^)				Anions (meq L^− 1^)		SAR (%)
			Ca^+ 2^	Mg^+ 2^	Na^+‎^	K^+‎^	HCO_− 3_‎	Cl^−^	
7.64	7.01	4486	18.14	17.98	49.73	0.036	4.22	51.60	11.70

**Fig. 1:**
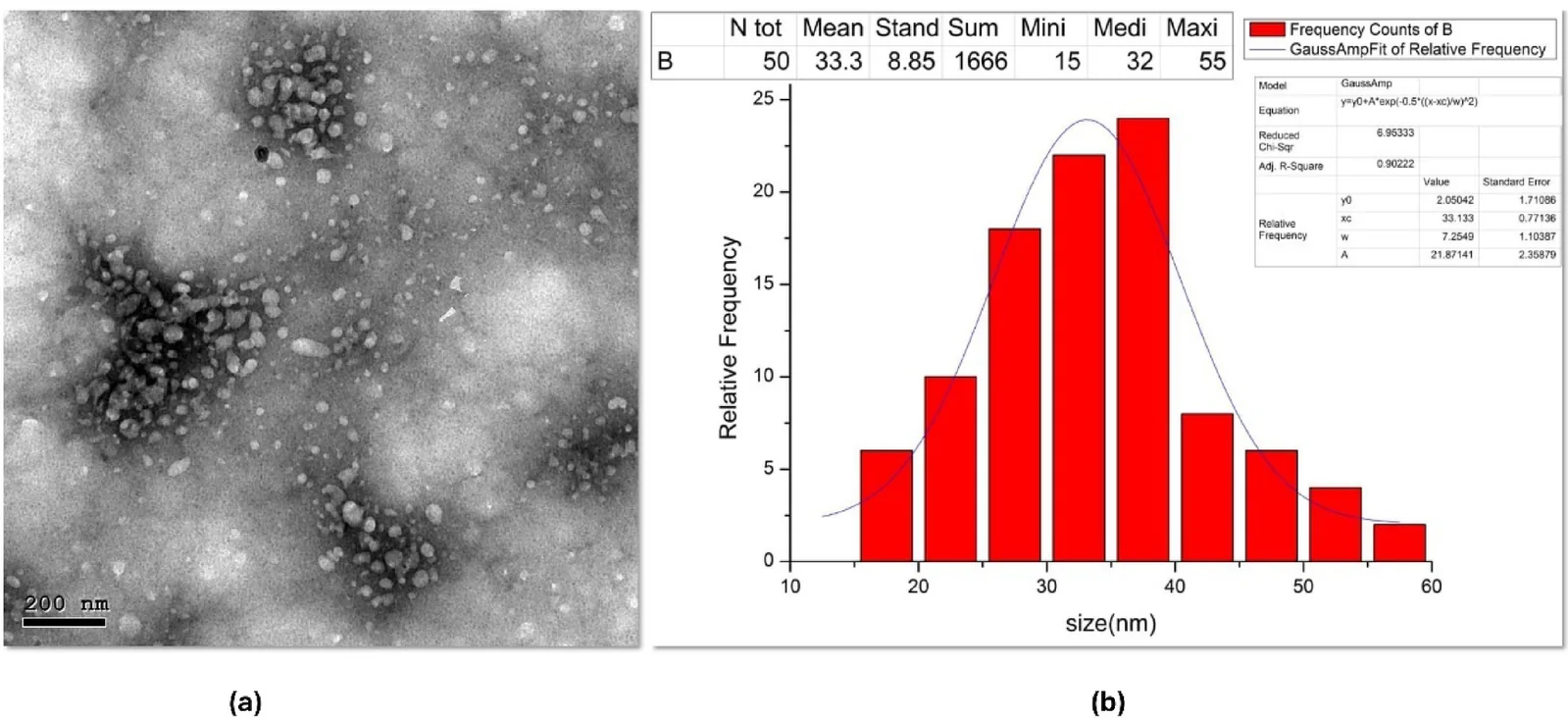
(**a**) Transmission electron microscopy (TEM) image of NPK nano-fertilizers (NPKNF), showcasing their structure and morphology. (**b**) Corresponding size distribution of the nanoparticles used in this study.

Twelve healthy ‘Picual’ olive trees, uniform in shape, size, and productivity, and subjected to the same horticultural practices including irrigation, soil fertilization, and pest management, were selected for the study. These trees were treated with NPK nano-fertilizers (NPKNF) at foliar application rates of 0 mg L^−1^ (control), 2000 mg L^−1^ (NPKNF-2000), 3000 mg L^−1^ (NPKNF-3000), and 4000 mg L^−1^ (NPKNF-4000). Control trees were sprayed with tap water. The NPKNF compound was purchased from nanotech company (http://www.nanotecheg.com/) located in 6th of October City, Cairo, Egypt, and was used according to the manufacturer’s instructions. The NPKNF was synthesized via an environmentally sustainable bottom-up alkylation method, optimized by the company for industrial-scale production. This method involved sequential crosslinking with dialkyl halide, followed by octyl halide alkylation and subsequent methylation using methyl iodide. Rutin-loaded chitosan nanoparticles were prepared by dissolving rutin in 70% ethanol, mixing it with chitosan, and incorporating nitrogen (N), phosphorus (P), and potassium (K) into the matrix. Nanoparticle formation was induced by the gradual addition of tripolyphosphate under continuous stirring, followed by two hours of agitation to ensure stabilization. The morphology and size of the synthesized NPKNF were also characterized by the company using transmission electron microscopy (TEM) (JEOL-JME 2100, Tokyo, Japan) as shown in Fig. 1.

NPKNF treatments were applied three times during the “on-year” season, specifically on July 1st, August 1st, and September 1st, to evaluate their effects on flowering and fruiting in the subsequent “off-year” season. Foliar sprays were applied at sunrise with an average spray solution volume of approximately 15 L per tree. Supplementary Data S1 provides a timeline of experimental activities, detailing procedures performed at each monthly interval.

### Measurements during the “on-year” season

In June, prior to the application of treatments, thirty healthy, fruit-bearing, unbranched one-year-old shoots were randomly selected and labelled on each tree, resulting in a total of 90 shoots per treatment. To ensure uniform spatial distribution, approximately seven shoots were chosen from each side of the canopy. The following measurements were subsequently performed on the designated shoots:

#### Vegetative growth characteristics

Vegetative growth characteristics were evaluated in the last week of October during the “on-year” season. Shoot length was measured pre- and post- spraying period (June and October 2022), with percentage increase calculated. The number of newly developed shoots emerging from each labelled shoot was recorded, and their length was measured using a ruler. Leaf fresh and dry weight were determined by collecting 10 leaves per replicate (30 leaves per treatment) from the fifth node of the shoot. Fresh weight was recorded immediately using a digital balance. For dry weight determination, after the samples were oven-dried at 70 °C for 72 h before weighing. Leaf area was assessed by collecting 30 mature leaves per treatment and calculated using the equation proposed by Ahmed and Morsy^51^: Leaf area (cm^2^) = 0.53 (Leaf length × Leaf width) + 1.66.

#### Leaf and flesh mineral contents

Leaf and fruit flesh mineral contents were analysed by collecting 10 leaves per replicate (30 leaves per treatment) from the fifth node of selected shoots in the last week of October. The samples were washed, dried, ground, and digested following Cottennie^52^. Total nitrogen was determined using the micro-Kjeldahl method^53^, potassium content was measured with a flame photometer^54^, and phosphorus was estimated following Jackson^55^. Similarly, during the fourth week of October in the “on-year” season, 30 fruits per treatment (10 per replicate) were randomly collected from all sides of the canopy. The fruit flesh was subsequently processed using the same digestion protocol employed for leaf samples.

#### Physical fruit characteristics

On the harvest date (last week of October), 10 fruits per replicate (30 fruits per treatment) were randomly collected from the treated trees. The following fruit parameters were assessed: fruit weight, flesh weight, and seed weight (g) were measured using a digital balance. Fruit volume (cm^2^) was measured using a measuring cylinder. Flesh thickness, fruit length, and fruit diameter were measured using a digital calliper. Flesh-to-seed ratio was calculated using the equation: Flesh/seed ratio = (Pulp weight (g))/(Seed weight (g)). Fruit shape index was determined using the equation: Fruit shape index = (Fruit length (cm))/(Fruit diameter (cm)).

#### Yield

Harvesting was carried out at the maturity stage during the last week of October, when approximately 75% of the olive fruits on each tree had developed a purple colouration. The total fruit yield per tree was individually weighed and recorded in kilograms (kg per tree).

#### Maturity index

A total of 100 olive fruits were randomly collected from each treatment, without replication for this specific parameter, and categorized into eight maturity stages based on skin and pulp coloration, as follows: (0) entirely green skin; (1) yellow-green skin; (2) red spots or discoloration covering less than 50% of the fruit surface; (3) red or light purple skin covering more than 50% of the fruit; (4) black skin with completely white pulp; (5) black skin with less than 50% of the pulp exhibitingpurple coloration; (6) black skin and more than 50% of the pulp showing purple coloration; and (7) black skin with fully purple pulp^56^. The maturity index (M.I.) was calculated using the following equation:

$${\mathrm{M}}{\mathrm{.I}}{\text{. = (A0 + B1 + C2 + D3 + E4 + F5 + G6 + H7)/100}}.$$

#### The moisture and oil content (%)

The moisture and oil content of ‘Picual’ olive fruits from all treatments, expressed on a dry matter basis, were determined according to the analytical procedures outlined by the Association of Official Analytical Chemists (AOAC)^57^.

#### Olive oil characteristics

Olive fruits (cv. ‘Picual’) from each treatment were crushed, and oil was extracted using a laboratory hydraulic press (Carver) operating at a gradual pressure of 12,000 lb/in^2^ for 30 min per sample. The resulting extract was transferred to a separatory funnel and allowed to settle to facilitate phase separation. The oil layer was then collected, centrifuged, and filtered through a cotton filter. The purified oil was stored in amber glass containers at − 20 °C until subsequent analysis.

The physicochemical properties of olive oil, including free fatty acids (%) as oleic acid, peroxide value (mg EqO_2_ kg^−1^ oil), saponification value (mg KOH/g oil) and unsaponifiable matter (%) were determined following the methods outlined by AOAC^57^. Additionally, the specific extinction coefficients at selected wavelengths (K_222_, K_270_, and ΔK) were assessed in accordance with IOC^58^. Total polyphenol content (mg/kg, expressed as caffeic acid) was extracted and quantified following the procedure described by Kouka et al.^59^. Antioxidant activity (%) of phenols extracts was determined using the stable 2, 2-diphenyl-1-picrylhydrazil (DPPH), as outlined by the same reference^59^. Fatty acid composition was determined by preparing fatty acid methyl esters (FAMEs) via cold transesterification using a potassium hydroxide methanolic solution. The FAMEs were subsequently analysed using an Agilent 6890 Series gas chromatograph (GC) equipped with a DB-23 capillary column (60 m × 0.32 mm), following the standard method outlined by ISO^60^. Chlorophyll and carotenoid contents (mg/kg of oil) were quantified spectrophotometrically using a UV-Spectrophotometer, according to the method described by Mínguez-Mosquera et al.^61^. Sensory evaluation of olive oils was conducted by a trained sensory panel following the official method for the organoleptic assessment of virgin olive oil^58^. The oils were assessed for key sensory attributes, including fruitiness, pungency, and bitterness, using an intensity scale ranging from 0 to 10. Final scores were calculated as the mean of the panelists’ assessments.

### Measurements during the “off-year” season

The same trees treated with NPKNF during the “on-year” season were monitored in the subsequent “off-year” season. In the last week of February, thirty healthy, unbranched 1-year-old shoots were randomly selected and labelled per tree, totalling 90 shoots per treatment. To ensure uniform spatial distribution, approximately seven shoots were selected from each side of the canopy. The following measurements were recorded on the labeled shoots:

#### Flowering parameters

Flowering parameters were assessed by evaluating the number of inflorescences per shoot, which was recorded as an average value. Flowering density was calculated as: Number of inflorescences per shoot/Shoot length (m). To assess the number of flowers per inflorescence and the percentage of perfect flowers, thirty inflorescences were randomly selected from pre-labelled shoots in each replicate. The average number of flowers per inflorescence was recorded, and the percentage of perfect flowers was calculated using the formula described by Fouad et al.^62^ as follows: perfect flowers (%) = (Number of perfect flowers/Number of total flowers per inflorescence) × 100.

#### Fruiting parameters

Fruit set, fruit drop, and yield were assessed based on standardized calculations. Initial fruit set percentage was determined three weeks after flowering (approximately 20 days after full bloom) using the equation by Fernandez-Escobar and Gomez-Valledor^63^: Initial fruit set (%) = (Number of fruit set/Shoot length (cm)) × 100. Final fruit set percentage was measured 60 days after full bloom using the same formula. Fruit drop percentage was calculated as: fruit drop (%) = [(Initial fruit set percentage - Final fruit set percentage) / Initial fruit set percentage) × 100.

#### Yield and alternate bearing severity

Yield assessment for the “off-year” season was conducted at the end of September when approximately 75% of the fruits on each tree had reached the purple maturity stage. The total yield per tree was recorded in kilograms per tree (kg per tree). To evaluate yield fluctuation between consecutive seasons, the alternate bearing index was calculated according to Monselise and Goldschmidt^64^ using the equation: Alternate bearing severity (%) = [(Yield of the “on-year” − Yield of the “off-year”)/Yield of the “on-year”] × 100. This index quantifies the degree of variation between high- and low-production years.

### Experiment design and statistical analysis

The experiment was designed as a randomized complete block design (RCBD), with each treatment comprising three replicates, and each replicate represented by a single tree. Data were subjected to analysis of variance (ANOVA) using R statistical package software version 4.4.1^65^. Model assumptions of normality were tested using the Shapiro–Wilk test (*P* ≤ 0.05) to validate the appropriateness of ANOVA. When these assumptions were not met, data were transformed to ensure that the residuals approximated a normal distribution. Statistical analyses were conducted using the normalized data; however, all results are presented using the original, untransformed values. Means comparisons were performed using Duncan’s multiple range tests at a 5% significance level^66^.

## Results and discussion

Data presented in Table 2 indicates a significant (*p* < 0.05) increase in ‘Picual’ shoot length rate, the number of new shoots per shoot, and the length of new shoots in response to NPK nano-fertilizers (NPKNF). The application of NPK at 4000 mg L^−1^ resulted in the highest values in shoot length increase rate (36.31%), number of new shoots per shoot (5.09), and new shoot length (6.04 cm). In contrast, the control treatment exhibited the lowest values for these parameters, with a shoot length increase rate of 24.85%, number of new shoots per shoot of 1.03, and a new shoot length of 3.04 cm. Additionally, all NPKNF treatments significantly (*p* < 0.05) increased leaf fresh and dry weights compared to the control. The 3000 and 4000 mg L^−1^ NPKNF treatments yielded the highest fresh weight (0.113 and 0.123 g, respectively) and dry weight (0.067 and 0.070 g, respectively), whereas the control treatment showed the lowest significant (*p* < 0.05) values for both fresh (0.087 g) and dry weight (0.047 g)., the leaf area was also positively influenced by the NPKNF treatments. The largest leaf area (3.88 cm^2^) was observed with the 4000 mg L^−1^ NPKNF treatment, while the control exhibited the smallest leaf area (3.23 cm^2^).

The observed increment in olive vegetative growth aligns with previous findings on date palm^34,35^, olive seedlings^49,50,67^, and Valencia orange^33^, which also reported significant improvements in vegetative growth following the application of NPKNF. The notable increase in growth characteristics following the application of NPK nano-fertilizers can be attributed to their unique properties, which facilitate deeper penetration into leaf tissues and promote efficient nutrient transport to the plant’s growth centers^68–70^. As a result, nutrient uptake is improved, leading to enhanced plant growth and development.

**Table 2:** Effect of spraying NPK nano-fertilizers on the vegetative growth characteristics of ‘Picual’ olive trees during the “on-year” season.

Treatments	Parameters					
	Increase in shoot length (%)	Number of new shoots per shoot	Length of new shoots (cm)	Leaf fresh weight (g)	Leaf dry weight (g)	Leaf area (cm^2^)
Control	24.85 ± 1.06 c	1.03 ± 0.05 d	3.04 ± 0.11 c	0.087 ± 0.01 b	0.047 ± 0.01 b	3.23 ± 0.05 c
NPKNF-2000	29.66 ± 1.05 b	2.13 ± 0.11 c	3.62 ± 0.18 c	0.110 ± 0.01 a	0.063 ± 0.00 a	3.60 ± 0.05 b
NPKNF-3000	33.44 ± 0.60 a	3.09 ± 0.12 b	4.54 ± 0.17 b	0.113 ± 0.01 a	0.067 ± 0.00 a	3.77 ± 0.02 a
NPKNF-4000	36.31 ± 0.63 a	5.09 ± 0.25 a	6.04 ± 0.25 a	0.123 ± 0.01 a	0.07 ± 0.00 a	3.88 ± 0.03 a

Values are presented as mean ± standard error (SE). Within each column, values followed by a common lowercase letter are not significantly different (Duncan’s Multiple Range Test, *p* < 0.05).

The role of NPK nutrients in promoting vegetative growth is well documented. Nitrogen (N) enhances photosynthesis, cell division, and elongation while also increasing meristematic activity by contributing to the synthesis of DNA, RNA, chlorophyll, amino acids, plant hormones (IAA), enzymes, and vitamins^8,29,69,71^. In addition, phosphorus (P) plays a crucial role in phospholipid synthesis, nucleic acids, and energy compounds (ATP), which enhance photosynthesis and respiration, ultimately leading to greater vegetative growth^29,68,69,71,72^. Furthermore, potassium (K) contributes significantly by regulating osmotic pressure, activating enzymes related to photosynthesis, and enhancing nutrient uptake, all of which improve plant vigor^29,68,69,71^. These combined effects of N, P, and K ensure an optimal balance of nutrients, leading to increased shoot length, number of new shoots, and enhanced leaf development.

The fresh and dry weight results of leaf agree with Hagagg et al.^67^who reported a significant impact of NPKNF on olive seedlings. The highest dry matter accumulation in treated olive leaves is associated with increased photosynthetic pigments, photosynthesis rates, and carbohydrate accumulation, which contribute to overall dry matter content^73^. This increased dry matter content enhances the plant’s ability to store and utilize essential resources, supporting overall productivity. Similarly, the increase in leaf area due to NPKNF application is consistent with the findings of Roshdy and Refaai^34^ on date palm, Mhawesh and Mohsen^50^ on olive seedlings, and El-Shereif et al.^33^ on Valencia orange. These studies demonstrated that NPKNF significantly enhances leaf expansion, thereby improving photosynthesis efficiency, which further contributes to increased vegetative growth. Table 3 presents data indicating that NPKNF treatments significantly (*p* < 0.05) enhanced leaf nitrogen, phosphorus, and potassium concentrations compared to the control. Among the tested treatments, the application of NPKNF at a concentration of 4000 mg L^−1^ resulted in the highest recorded levels of leaf nitrogen (2.85%), phosphorus (0.44%), and potassium (1.44%).

In contrast, the control exhibited the lowest concentrations, with values of 2.28% for nitrogen, 0.016% for phosphorus, and 0.80% for potassium. Similarly, the data in Table 3 demonstrate that NPKNF treatments had a favourable impact on the nutrient composition of the fruit flesh. The highest nitrogen (0.84%), phosphorus (0.36%), and potassium (2.93%) concentrations in the flesh were observed in response to NPKNF application at 4000 mg L^−1^. Conversely, the control treatment resulted in the lowest recorded values, with nitrogen, phosphorus, and potassium concentrations of 0.73%, 0.24%, and 2.29%, respectively. Such improvements in leaf nutrient composition following NPKNF application have been observed in various horticultural species, including date palm (*Phoenix dactylifera*)^34,35^, mango (*Mangifera indica*)^37–39^, and orange (*Citrus sinensis*)^33^. The observed increase in leaf mineral content may be attributed to the role of nanoelements in enhancing vegetative growth and metabolic activity, thereby promoting the accumulation of essential chemical constituents in leaf tissues^69,74^.

**Table 3:** Effect of spraying NPK nano-fertilizers on leaf and fruit N, P, and K content of ‘Picual’ olive trees during the “on-year” season.

Treatments	Parameters					
	Leaf			Flesh		
	N (%)	P (%)	K (%)	N (%)	P (%)	K (%)
Control	2.28 ± 0.05 c	0.16 ± 0.04 c	0.80 ± 0.08 c	0.73 ± 0.02 b	0.24 ± 0.03 b	2.29 ± 0.04 c
NPKNF-2000	2.65 ± 0.05 b	0.22 ± 0.01 bc	0.99 ± 0.02 bc	0.76 ± 0.01 ab	0.33 ± 0.02 a	2.35 ± 0. 06 c
NPKNF-3000	2.79 ± 0.06 ab	0.31 ± 0.04 b	1.18 ± 0.08 b	0.81 ± 0.02 ab	0.34 ± 0.04 a	2.64 ± 0.05 b
NPKNF-4000	2.85 ± 0.05 a	0.44 ± 0.01 a	1.44 ± 0.02 a	0.84 ± 0.04 a	0.36 ± 0.01 a	2.93 ± 0.08 a

Values are presented as mean ± standard error (SE). Within each column, values followed by a common lowercase letter are not significantly different (Duncan’s Multiple Range Test, *p* < 0.05).

The data presented in Table 4 demonstrate that fruit weight increased significantly (*p* < 0.05) in response to the foliar applications of NPKNF compared to the control. The highest fruit weight was recorded in trees treated with NPKNF at 4000 mg L^−1^ (6.94 g), followed by those treated with NPKNF at 3000 mg L^−1^ (6.47 g). The lowest fruit weight was observed in the control treatment (5.34 g). Additionally, all NPKNF treatments had a statistically significant impact on fruit volume. Trees sprayed with NPKNF at 4000 mg L^−1^ exhibited the highest fruit volume (6.09 cm^2^), followed by those treated with NPKNF at 3000 mg L^−1^ (5.40 cm^2^), whereas the control treatment resulted in the lowest recorded fruit volume (4.12 cm^2^). In contrast to fruit weight and volume, flesh thickness did not exhibit significant (*p* < 0.05) differences among treatments, as indicated by the data in Table 4. However, flesh weight was notably influenced by NPKNF application, with 4000 mg L^−1^ treatment yielding the highest recorded value (6.11 g), while the control treatment resulted in the lowest flesh weight (4.52 g). Regarding seed weight, no significant (*p* < 0.05) differences were observed among the treatments. Nevertheless, the pulp-to-seed ratio was significantly (*p* < 0.05) affected by the applied treatments, with the highest ratio recorded in the 4000 mg L^−1^ NPKNF treatment (7.56), whereas the control treatment exhibited the lowest ratio (5.52). The findings of the present study regarding fruit weight align with those of El-Shereif et al.^33^, who observed that NPKNF application enhanced fruit weight in Valencia orange.

**Table 4:** Effect of spraying NPK nano-fertilizers on physical fruit characteristics of ‘Picual’ olive trees during the “on-year” season.

Treatments	Parameters					
	Fruit weight (g)	Fruit volume (cm^3^)	Flesh thickness (mm)	Flesh weight (g)	Seed weight (g)	Flesh/seed ratio
Control	5.34 ± 0.28 c	4.12 ± 0.21 c	4.98 ± 0.22 a	4.52 ± 0.23 c	0.82 ± 0.05 a	5.52 ± 0.12 b
NPKNF-2000	5.81 ± 0.29 bc	5.00 ± 0.20 b	5.12 ± 0.20 a	5.00 ± 0.23 bc	0.81 ± 0.08 a	6.23 ± 0.32 b
NPKNF-3000	6.47 ± 0.32 ab	5.40 ± 0.26 ab	5.60 ± 0.19 a	5.59 ± 0.22 ab	0.88 ± 0.09 a	6.46 ± 0.51 ab
NPKNF-4000	6.94 ± 0.35 a	6.09 ± 0.23 a	5.78 ± 0.24 a	6.11 ± 0.24 a	0.83 ± 0.11 a	7.56 ± 0.72 a

Values are presented as mean ± standard error (SE). Within each column, values followed by a common lowercase letter are not significantly different (Duncan’s Multiple Range Test, *p* < 0.05).

Similarly, Gad et al.^75^ demonstrated that foliar spraying with nano-potassium silicate significantly increased fruit weight in the ‘Ewais’ mango cultivar compared to the untreated control. Roshdy and Refaai^34^ also reported that NPKNF application enhanced the average fruit weight of date palm. The significant increase in fruit volume observed in our study aligns with the findings of Morales-Sillero et al.^76^, who reported that NPK fertilizer application improved fruit volume in mature ‘Manzanillo’ olive cultivar. Inglese et al.^77^ noted that nitrogen and potassium fertilization during the pit-hardening stage increased fruit volume. Our results on flesh weight corroborate those of Abbasi et al.^78^, who found that combined foliar application of micro- and macronutrients significantly improved olive flesh weight.

Furthermore, the present findings regarding the pulp-to-seed ratio are consistent with Roshdy and Refaai^34^, who reported that NPKNF significantly increased the pulp percentage in date palm. Ben Mimoun et al.^79^ also demonstrated that potassium fertilization improved yield and fruit quality, including fruit weight and the flesh (pulp)-to-seed ratio. Several other studies have similarly reported that NPK fertilization enhances both fruit weight and the pulp-to-seed ratio^76–78,80^. The use of nano-based NPK fertilizers has been shown to significantly improve fruit tree productivity^75,81^. This improvement in fruit characteristics may be attributed to the ability of nano-fertilizers to boost photosynthetic efficiency, thereby increasing photoassimilate production and their subsequent translocation and accumulation in fruits^82,83^. Additionally, elevated nutrient concentrations in leaves may substantially influence fruit growth and overall yield^75,84^.

The data in Table 5 indicate that the applied treatments had no statistically significant effect on fruit length, diameter, or shape index. Similarly, yield measurements showed no significant differences between NPKNF-treated trees and the control.

**Table 5:** Effect of spraying NPK nano-fertilizers on physical fruit characteristics and yield of ‘Picual’ olive trees during the “on-year” season.

Treatments	Parameters			
	Fruit length (cm)	Fruit diameter (cm)	Fruit shape index (L/D)	Yield (kg per tree)
Control	2.35 ± 0.09 a	1.83 ± 0.08 a	1.28 ± 0.00 a	47.67 ± 1.50 a
NPKNF-2000	2.46 ± 0.11 a	1.92 ± 0.06 a	1.28 ± 0.02 a	48.00 ± 2.09 a
NPKNF-3000	2.36 ± 0.09 a	1.94 ± 0.06 a	1.22 ± 0.01 a	50.67 ± 2.03 a
NPKNF-4000	2.39 ± 0.09 a	2.00 ± 0.05 a	1.19 ± 0.04 a	51.33 ± 0.88 a

Values are presented as mean ± standard error (SE). Within each column, values followed by a common lowercase letter are not significantly different (Duncan’s Multiple Range Test, *p* < 0.05).

The maturity index of ‘Picual’ olive fruits was determined based on skin and flesh colour, following the methodology described in the materials and methods section, using 100 fruits per treatment. Supplementary Figure S1 indicates that fertilization with the NPKNF delayed fruit ripening. These findings are consistent with those reported by Rodrigues et al.^85^. However, they contrast with the results of Rosati et al.^86^who concluded that fertilization did not affect fruit maturation.

**Fig. 2:**
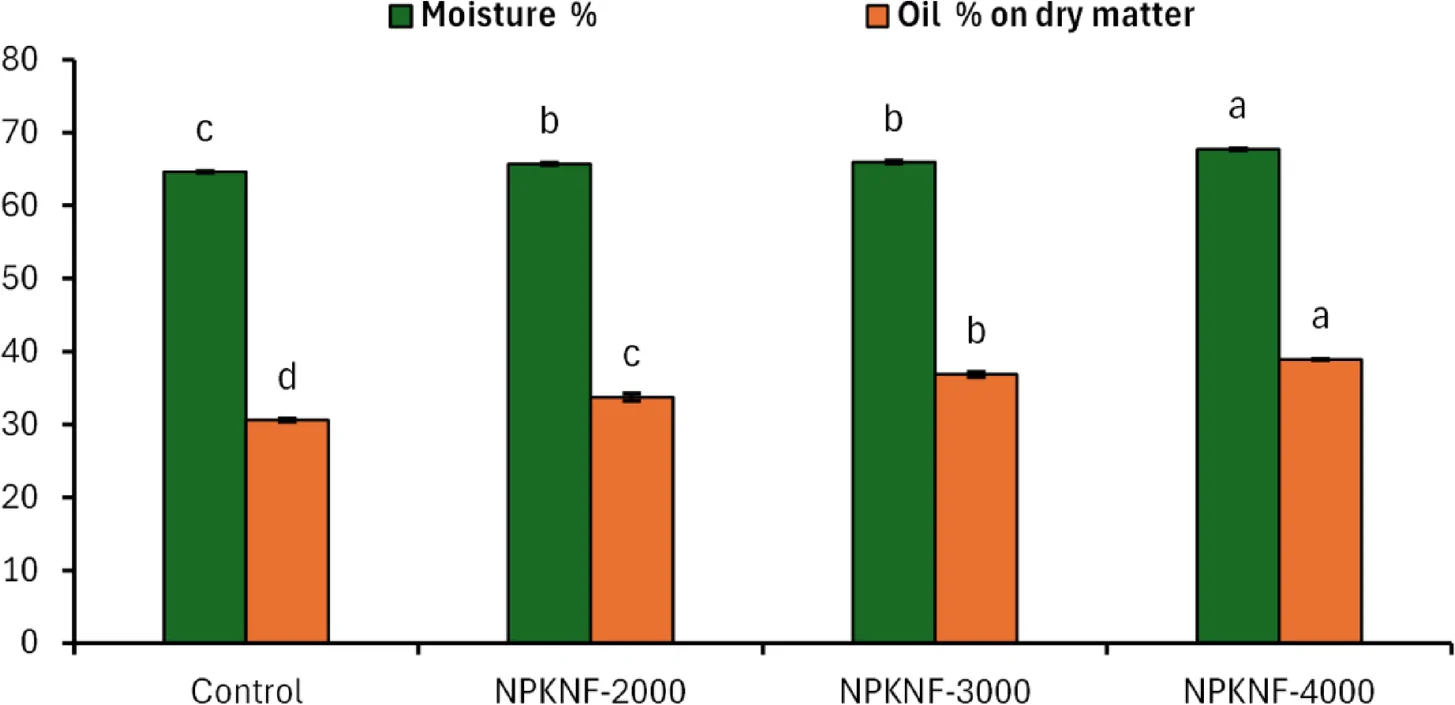
Effect of spraying NPK nano-fertilizers on moisture and oil % of ‘Picual’ olive trees during the “on-year” season. Identical letters within the same trait indicate the absence of statistically significant differences between treatments at the 5% significance level. The error bar represents the standard error (± SE).

The moisture and oil content (%) of ‘Picual’ olive fruits were influenced by NPKNF fertilization (Fig. 2). The results indicated that increasing the concentration of the NPKNF led to a corresponding increase in both oil and moisture content compared to unfertilized fruits (control). The oil content (on a dry weight basis) increased from 30.8% in unfertilized olive fruits to 38.9% in fruits treated with the NPKNF at a concentration of 4000 mg L^−1^.

These findings align with those of Rosati et al.^86^, who reported that foliar fertilization with potassium and nitrogen enhances oil and moisture content in olive fruits. Fertilization typically increases both the pulp-to-stone ratio and oil content. Since the pulp contains significantly more oil than the stone, an increase in the pulp-to-stone ratio leads to higher overall oil content without altering the oil concentration within the pulp itself. Consequently, the commonly reported increase in oil content associated with fertilization can be attributed to the enhanced pulp-to-stone ratio rather than a direct increase in the oil concentration of the pulp^86^.

Olive oil classification (extra virgin, virgin, ordinary, and lampante) is determined through chemical analysis of free fatty acids (FFA), peroxide value (PV), ultraviolet absorption coefficients (K_222_ and K_270_), and ΔK, along with sensory evaluation^58^. As shown in Table 6, the FFA values ranged from 0.08 to 0.11% as oleic acid, while PV varied between 5.03 and 5.85 meq O₂/kg oil. The ultraviolet absorption values at K_222_ and K_270_ ranged from 0.913 to 1.049 and from 0.055 to 0.064, respectively. Importantly, all oil samples, including those from both treated and untreated fruits, exhibited FFA, PV and UV absorbance (at 232 and 270 nm) and ∆K values that were below the maximum limits established by the International Olive Council (IOC) for extra virgin olive oil^58^. These results align with the findings of Zipori et al.^83^, Busso et al.^87^, Tekaya et al.^88^, and, who reported that while proper fertilization improves tree physiology, photosynthesis, and productivity, its influence on oil quality parameters appears limited. The saponification values, which reflect average fatty acid chain length, were determined to be 193.27 mg KOH/g oil for the untreated control, and 193.22, 193.26, and 193.33 mg KOH/g oil for oils from fruits treated with NPKNF at concentrations of 2000, 3000, and 4000 mg L^−1^, respectively, all of which comply with both Codex^89^ and Egyptian^90^ standards. Furthermore, the phenolic content analysis revealed that foliar fertilization resulted in reduced total polyphenol content (expressed as caffeic acid) compared to the control (350.51 mg/kg), with values decreasing to 298.59, 289.28, and 277.11 mg/kg for oils from fruits treated with 2000, 3000, and 4000 mg L^−1^ NPKNF, respectively. Since phenolic compounds significantly contribute to olive oil’s nutritional value, sensory characteristics, and oxidative stability^91–95^, this observed reduction in polyphenol content aligns with findings from previous studies^94,96^.

The observed reduction in phenolic content following fertilization may result from altered activity of key enzymes including phenylalanine ammonia-lyase (PAL), polyphenol oxidase (PPO), and peroxidase (POD), which regulate phenolic compound biosynthesis. PAL plays a crucial role in this process by catalysing the conversion of phenylalanine to cinnamic acid, thereby directing carbon flow from primary to secondary metabolism through the phenylpropanoid pathway. This enzymatic reaction also serves an important function in nitrogen metabolism by liberating nitrogen from phenylalanine during its transformation to cinnamic acid, facilitating nitrogen redistribution particularly under nitrogen-limited conditions. Following nitrogen fertilization, competition may arise between protein synthesis and phenolic compound biosynthesis for their common precursor, phenylalanine. In such cases, phenylalanine is likely preferentially allocated to protein synthesis rather than being diverted into the phenylpropanoid pathway via PAL activity, consequently restricting phenylpropanoid biosynthesis. This metabolic competition provides a plausible explanation for the decreased phenolic content observed with nitrogen fertilization^95,96^. Supporting these biochemical observations, antioxidant activity assessments using the 2,2-diphenyl-1-picrylhydrazyl (DPPH) free radical assay revealed that phenolic extracts from oil obtained from unfertilized fruits exhibited superior antioxidant capacity compared to those derived from NPKNF-treated olives, as detailed in Table 6. The observed antioxidant activity demonstrates a positive correlation with total phenolic content^91,92^consistent with established biochemical relationships. Unsaponifiable matter (UNSAP), comprising hydrocarbons, long-chain alcohols, fat-soluble vitamins, and phytosterols, represents the oil fraction resistant to alkaline saponification but soluble in nonpolar solvents. This component serves as a critical marker for olive oil authenticity verification^58^. All analysed oil samples showed UNSAP percentages below the International Olive Council’s 1.5% threshold^58^, with values progressively increasing alongside NPKNF concentration and peaking in the 4000 mg L^−1^ treatment group. Our findings align with those of Rosati et al.^86^, who demonstrated that foliar nitrogen fertilization enhances the nutritional and health value of olive oil by increasing the levels of bioactive compounds, such as phytosterols. However, contrasting findings by Jordão et al.^97^ showed no significant impact of combined nitrogen, phosphorus, and calcium foliar applications on phytosterol content.

**Table 6:** Quality characteristics of ‘picual’ Olive oil in response to foliar fertilization with a nano mixture of NPK during the “on-year” season.

Parameters		Treatments			
		Control	NPKNF-2000	NPKNF-3000	NPKNF-4000
Free fatty acids % as oleic acid		0.08 ± 0.00 b	0.08 ± 0.00 b	0.09 ± 0.00 ab	0.11 ± 0.01 a
Peroxide value (mg EqO_2_ kg^−1^ oil)		5.85 ± 0.17 a	5.03 ± 0.10 d	5.27 ± 0.16 c	5.69 ± 0.19 b
K_232_		0.96 ± 0.01 c	0.913 ± 0.00 d	1.021 ± 0.00 b	1.049 ± 0.00 a
K_270_		0.064 ± 0.00 a	0.05 5± 0.00 d	0.057 ± 0.00 c	0.062 ± 0.00 b
ΔK		-0.0035 ± 0.00 b	-0.0015 ± 0.00 a	-0.004 ± 0.00 b	-0.0017 ± 0.00 a
Saponification value (mg KOH/g oil)		193.27 ± 0.09 a	193.22 ± 0.05 a	193.26 ± 0.03 a	193.33 ± 0.01 a
Total poly phenols (mg/kg)		350.51 ± 2.99 a	298.59 ± 4.27 b	289.28 ± 0.91 b	277.11 ± 1.66 c
Antioxidant activity of phenols extract (%)		61.92 ± 0.66 a	58.42 ± 0.07 b	53.16 ± 0.12 c	50.84 ± 0.61 d
Unsaponifiable matters (%)		1.01 ± 0.04 c	1.19 ± 0.01 b	1.23 ± 0.02 ab	1.3 ± 0.02 a
Pigments (mg/kg)	Chlorophylls	1.8± 0.02 d	1.98 ± 0.06 c	2.18 ± 0.02 b	2.39 ± 0.02 a
	Carotenoids	1.53 ± 0.02 c	1.74 ± 0.03 b	1.81 ± 0.02 ab	1.89 ± 0.03 a

Values are presented as mean ± standard error (SE). Within each row, values followed by a common lowercase letter are not significantly different (Duncan’s Multiple Range Test, *p* < 0.05).

Pigment composition substantially affects olive oil quality, influencing both visual characteristics (a primary consumer evaluation criterion) and oxidative stability. Chlorophylls and carotenoids, the primary pigments, measured 1.80 and 1.53 mg/kg, respectively, in control samples, increasing dose-dependently with NPKNF treatment to maximum concentrations of 2.39 and 1.89 mg/kg at 4000 mg L^−1^.

Chromatographic analysis (Table 7) revealed a characteristic olive oil fatty acid profile spanning C14 to C22 chains, with oleic acid (C18:1) as the predominant component. The chromatographic analysis revealed significant treatment effects on fatty acid composition. Oleic acid (C18:1) content showed a concentration-dependent increase with NPKNF application, rising from 63.50% in the control to 63.83%, 63.89%, and 64.06% at 2000, 3000, and 4000 mg L^−1^, respectively.

**Table 7:** Relative percentage of fatty acids of ‘Picual’ olive oil in response to foliar fertilization with a nano mixture of NPK during the “on-year” season.

Relative percentage of fatty acids (%)	Control	NPKNF-2000	NPKNF-3000	NPKNF-4000
Myristic acid (C14:0)	0.25	0.03	0.02	0.03
Palmitic acid (C16:0)	18.79	19.09	19.32	19.49
Palmitoleic acid (C16:1)	3.32	3.23	3.19	3.12
Maragic acid (C17:0)	0.08	0.05	0.06	0.04
Margoleic acid (C17:1)	0.18	0.16	0.15	0.10
Stearic acid (C18:0)	2.22	2.29	2.31	2.34
Oleic acid (C18:1)	63.50	63.83	63.89	64.06
Linoleic acid (C18:2)	9.83	9.67	9.55	9.40
Linolenic acid (C18:3)	1.07	1.00	0.93	0.90
Arachidic acid (C20:0)	0.37	0.33	0.30	0.29
Gadoleic acid (C20:1)	0.25	0.21	0.19	0.17
Behenic acid (C22:0)	0.14	0.11	0.09	0.06
C18:1/C18:2	6.46	6.60	6.69	6.81
Saturated fatty acids	21.85	21.90	22.10	22.25
Unsaturated fatty acids	78.15	78.10	77.90	77.75
Monounsaturated fatty acids (MUFA)	67.25	67.43	67.42	67.45
Polyunsaturated fatty acids (PUFA)	10.90	10.67	10.48	10.30
MUFA/PUFA ratio	6.17	6.32	6.43	6.55
Iodine value (g I_2_ 100 g^−1^ oil)	81.04	80.98	80.57	80.28

**Fig. 3:**
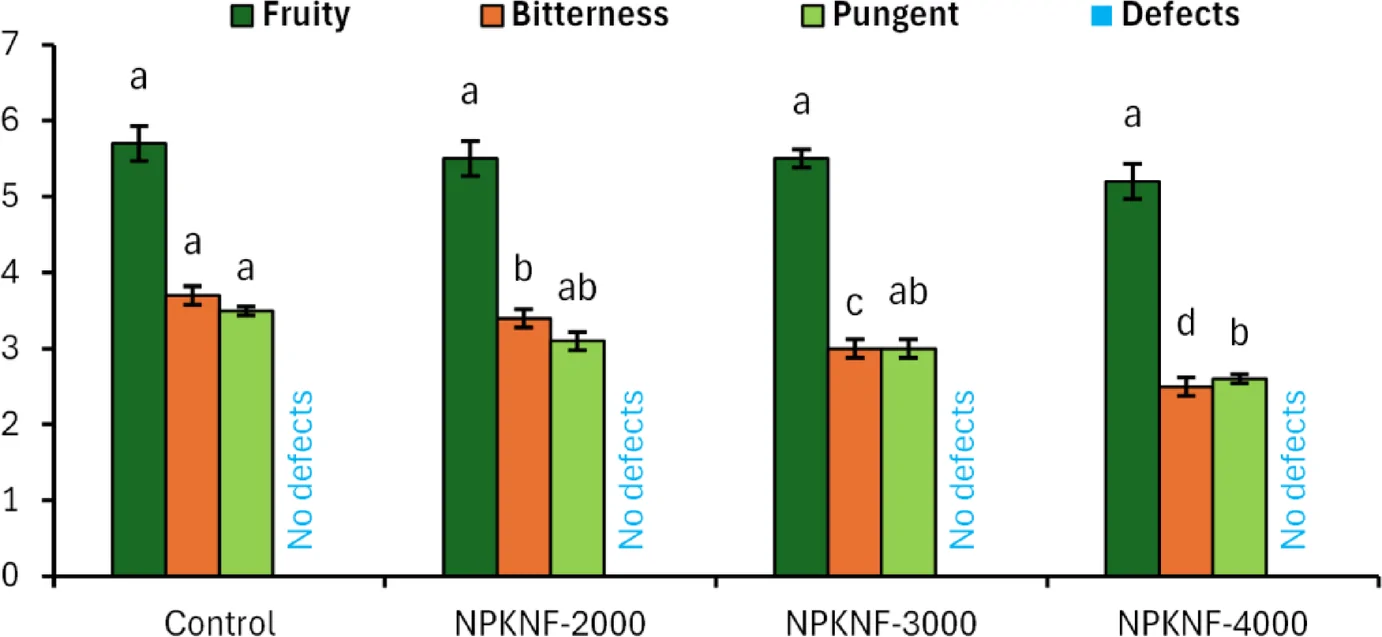
Effect of spraying NPK nano-fertilizers on sensory evaluation of ‘Picual’ olive trees during the “on-year” season. Identical letters within the same trait indicate the absence of statistically significant differences between treatments at the 5% significance level. The error bar represents the standard error (± SE).

Conversely, the linolenic acid (C18:3) content decreased progressively with fertilization intensity, measuring 1.00%, 0.93%, and 0.90% in treated samples (2000, 3000, and 4000 mg L^−1^, respectively) compared to 1.07% in the control. Notably, the control value exceeded the International Olive Council’s maximum limit of 1.0% for linolenic acid^58^. The NPKNF treatment elevated the total monounsaturated fatty acid (MUFA) content, primarily by increasing oleic acid (C18:1) concentration. Conversely, it reduced the total polyunsaturated fatty acid (PUFA) content, specifically diminishing linoleic (C18:2) and linolenic (C18:3) acids. This shift resulted in a higher MUFA/PUFA ratio than in the control. This rebalancing towards a more monounsaturated profile is known to confer greater resistance to oxidative degradation and rancidity. Calculated iodine values, reflecting the oil’s degree of unsaturation, ranged from 80.28 to 81.04 g iodine/100 g oil across all samples. These variations in iodine value directly correlate with the observed changes in unsaturated fatty acids, particularly oleic (C18:1) and linoleic (C18:2) acids, demonstrating the influence of foliar fertilization on the oil’s fatty acid profile. The sensory profile of olive oil comprises both positive and negative attributes as defined by IOC^58^. Positive characteristics include fruitiness, bitterness, and pungency, while negative attributes or defects encompass musty, fusty, winey, rancid, metallic, and muddy sediment notes. Phenolic compounds significantly influence this sensory profile, particularly by enhancing bitterness and pungency^91–93^. As presented in Fig. 3, fertilization treatment did not significantly affect fruitiness intensity. However, the unfertilized control sample demonstrated notably higher bitterness and pungency scores compared to fertilized samples, consistent with its higher phenolic content. Importantly, all evaluated oil samples satisfied all requirements for extra virgin olive oil classification according to IOC^58^ standards. The classification was validated based on two main criteria. First, the median intensity of positive sensory attributes remained above zero in all samples. Second, no sensory defects were observed. These findings indicate that NPKNF fertilization may influence specific sensory attributes without compromising the oil’s classification as extra virgin olive oil.

Regarding the measurements taken during the “off-year” season, data from Table 8 reveal that foliar application of NPKNF significantly increased the number of inflorescences per shoot. The most pronounced effect was observed at a concentration of 4000 mg L^−1^, which yielded the highest recorded value of 8.76. In contrast, the control treatment exhibited the lowest number of inflorescences per shoot (3.53). Additionally, Table 8 demonstrates that all tested NPKNF treatments significantly enhanced flowering density compared to the control. The highest flowering density (31.73) was observed in trees treated with NPKNF at 4000 mg L^−1^, whereas the control recorded the lowest value (16.86). Furthermore, the data indicates that NPKNF application had a significant positive effect on the number of flowers per inflorescence. The highest number of flowers per inflorescence (13.34) was observed in response to NPKNF application at 4000 mg L^−1^, while the control treatment recorded the lowest value (7.72). However, with respect to the percentage of perfect flowers, no significant differences were detected among the treatments, as shown in Table 8.

The observed increase in flowering intensity aligns with the findings of Erel et al.^16^who reported that the availability of nitrogen, phosphorus, and potassium influences the number of inflorescences per shoot in olive trees. This improvement in flowering characteristics may be attributed to the role of NPK nano-fertilizers in enhancing chlorophyll content and photosynthetic activity, thereby promoting flower development^75^.

**Table 8:** Effect of spraying NPK nano-fertilizers on flowering parameters of ‘Picual’ olive trees during the “off-year” season.

Treatments	Parameters			
	No. infl./shoot	Flowering density	No. flowers/infl.	Perfect flower (%)
Control	3.53 ± 0.78 c	16.86 ± 1.63 c	7.72 ± 1.01 b	42.83 ± 1.30 a
NPKNF-2000	5.95 ± 1.01 b	26.96 ± 1.09 b	10.48 ± 1.29 ab	44.56 ± 1.37 a
NPKNF-3000	7.12 ± 0.71 ab	27.26 ± 1. 47 b	11.31 ± 1.01 a	45.25 ± 1.09 a
NPKNF-4000	8.76 ± 0.46 a	31.73 ± 0.76 a	13.34 ± 0.95 a	45.54 ± 1.56 a

Values are presented as mean ± standard error (SE). Within each column, values followed by a common lowercase letter are not significantly different (Duncan’s Multiple Range Test, *p* < 0.05).

The data presented in Table 9 indicate that the applied treatments did not result in statistically significant differences in the initial fruit set percentage. However, a significant and progressive increase in the final fruit set percentage was observed, with the highest recorded value of 18.6% at the highest NPKNF concentration. Furthermore, the results demonstrate significant differences among treatments in terms of fruit drop percentage. The control exhibited the highest fruit drop rate (53.31%), whereas the lowest percentage (37.01%) was recorded in trees treated with NPKNF at 4000 mg L^−1^. Regarding yield, Table 9 also shows a significant increase in yield (kg per tree) in response to NPKNF treatments. The highest yield (7.5 kg per tree) was obtained with the application of NPKNF at 4000 mg L^−1^, while the control treatment resulted in the lowest yield (3.5 kg per tree). Additionally, the data indicates that alternate bearing severity decreased significantly with increasing NPKNF concentrations. The lowest alternate bearing severity (85.4) was observed in trees treated with NPKNF at 4000 mg L^−1^. The results clearly demonstrate that NPKNF application enhanced the final fruit set and yield of ‘Picual’ olive trees while reducing fruit drop and improving fruit retention. These findings are consistent with those of Gad et al.^75^, who reported that foliar application of nano-potassium silicate significantly improved the final fruit set, yield, and fruit retention in the ‘Ewais’ mango cultivar. Similarly, Saied^37^ observed an increase in yield and fruit retention in mango following the application of NPKNF. Davarpanah et al.^40^ also reported that nano-nitrogen application in pomegranate led to increased fruit yield. The observed improvements in fruit yield and retention may be attributed to enhanced nutrient concentrations in the leaves, which have a substantial influence on fruit growth and overall productivity^75,84^.

**Table 9:** Effect of spraying NPK nano-fertilizers on fruiting parameters, yield (kg per tree) and alternate bearing severity of ‘Picual’ olive trees during the “off-year” season.

Treatments	Parameters				
	Initial fruit set (%)	Final fruit set (%)	Fruit drop (%)	Yield (kg per tree)	Alternate bearing severity
Control	26.24 ± 1.56 a	12.23 ± 0.56 c	53.31 ± 0.66 a	3.50 ± 0.14 c	92.66 ± 0.09 a
NPKNF-2000	27.33 ± 1.61 a	15.09 ± 1.39 bc	44.96 ± 2.26 b	5.33 ± 0.44 b	88.92 ± 0.54 b
NPKNF-3000	28.07 ± 1.37 a	16.75 ± 0.81 ab	40.31 ± 0.68 bc	6.25 ± 0.43 ab	87.70 ± 0.36 b
NPKNF-4000	29.40 ± 1.83 a	18.60 ± 1. 84 a	37.01 ± 2.37 c	7.50 ± 0.29 a	85.40 ± 0.32 c

Values are presented as mean ± standard error (SE). Within each column, values followed by a common lowercase letter are not significantly different (Duncan’s Multiple Range Test, *p* < 0.05).

Generally, the targeted application of nano-fertilizers demonstrates considerable promise in enhancing crop performance through improved nutrient delivery and uptake. Across various crops, including wheat^98,99^, maize^99^, tomato^100^, and soybean^101^, nano-fertilizers have been associated with increased enzyme activity, enhanced photosynthetic efficiency, and more effective nutrient transport. These physiological and metabolic improvements collectively support healthier plant development and higher productivity. The observed benefits highlight the potential of nano-fertilizers as a viable strategy for advancing sustainable agricultural practices.

Furthermore, the findings indicate that NPKNF application effectively reduced alternate bearing severity. This result aligns with the studies of Kotsias et al. and Haberman et al.^19,20^, which reported that nitrogen fertilization mitigates the phenomenon of alternate bearing in olive trees. The observed increase in olive yield during the “off-year” and the reduction in alternate bearing severity may be due to the role of NPKNF in promoting the development of new shoots in the preceding “on-year”. Since olive trees bear fruit on one-year-old shoots, an increased number of new shoots in the “on-year” positively impacts flowering and fruiting in the subsequent “off-year”.

## Conclusion

This study investigated the effects of foliar application of the green synthesis NPK nano-fertilizers (NPKNF) on ‘Picual’ olive trees during the “on-year” season, evaluating their impact on vegetative growth, flowering, fruit set, yield, and olive oil quality. The application of NPKNF at a concentration of 4000 mg L^− 1^ resulted in significant improvements in all measured parameters compared to untreated trees. Furthermore, in the subsequent “off-year” season, the treatments enhanced flowering and yield while reducing the severity of alternate bearing. These results underscore the effectiveness of NPKNF, particularly when applied foliarly at a concentration of 4000 mg L^− 1^, in enhancing olive tree productivity under alternate bearing conditions. This study is limited by its focus on a single olive cultivar, which may not capture the variability in alternate bearing among different genotypes. Future research should include a broader range of cultivars, especially those showing pronounced biennial bearing, and incorporate biochemical and physiological analyses, such as nutrient uptake and enzymatic activity, to better understand the underlying mechanisms. Finally, this study paves the way for future efforts on other crops, such as mango trees, which also exhibit a strong tendency toward alternate bearing.

## Supplementary Information

Below is the link to the electronic supplementary material.

Supplementary Material 1

Supplementary Material 2

Supplementary Material 3

## Data Availability

The data generated and/or analysed during the current study are available from the corresponding author on reasonable request.
