# Supplementary material for: NPK nano-fertilizers enhance growth, oil quality, and yield regularity in Picual olive trees
Source: Sci Rep. 2025 Sep 12;15:32495. doi: 10.1038/s41598-025-17267-9 (PMC12432146; doi:10.1038/s41598-025-17267-9)
Supplement: Supplementary file 2 — Supplementary Material 2 [file 41598_2025_17267_MOESM2_ESM.docx]

**Climate Report (2022–2024)**

**2022 — Daily-Yearly Climate Averages**

| **Parameter** | **Approx. Value** |
| --- | --- |
| **Max Temperature (°C)** | **39 °C** |
| **Min Temperature (°C)** | **11 °C** |
| **Average Temperature (°C)** | **25 °C (daily mean)** |
| **Relative Humidity (%)** | **30 % annual average** |
| **Annual Precipitation (mm)** | **5 mm** |

**2023 — Daily-Yearly Climate Averages**

| **Parameter** | **Approx. Value** |
| --- | --- |
| **Max Temperature (°C)** | **40 °C** |
| **Min Temperature (°C)** | **12 °C** |
| **Average Temperature (°C)** | **26 °C** |
| **Relative Humidity (%)** | **30–35 %** |
| **Annual Precipitation (mm)** | **5 mm** |

**2024 — Daily-Yearly Climate Averages**

| **Parameter** | **Approx. Value** |
| --- | --- |
| **Max Temperature (°C)** | **40–41 °C** |
| **Min Temperature (°C)** | **12–13 °C** |
| **Average Temperature (°C)** | **26–27 °C** |
| **Relative Humidity (%)** | **30–35 %** |
| **Annual Precipitation (mm)** | **5 mm** |

**2022 — Monthly Climate Averages**

| **Parameter** | **January** | **February** | **March** | **April** | **May** | **June** | **July** | **August** | **September** | **October** | **November** | **December** |
| --- | --- | --- | --- | --- | --- | --- | --- | --- | --- | --- | --- | --- |
| **Max Temp (°C)** | **18** | **20** | **24** | **29** | **34** | **37** | **39** | **39** | **35** | **30** | **24** | **19** |
| **Min Temp (°C)** | **6** | **7** | **10** | **14** | **19** | **22** | **24** | **24** | **21** | **17** | **11** | **7** |
| **Avg Temp (°C)** | **12** | **14** | **17** | **22** | **27** | **30** | **32** | **32** | **28** | **24** | **18** | **13** |
| **Humidity (%)** | **60** | **55** | **50** | **40** | **30** | **25** | **25** | **25** | **30** | **40** | **50** | **60** |
| **Rainfall (mm)** | **1** | **1** | **0** | **0** | **0** | **0** | **0** | **0** | **0** | **1** | **1** | **1** |

**2023 — Monthly Climate Averages**

| **Parameter** | **January** | **February** | **March** | **April** | **May** | **June** | **July** | **August** | **September** | **October** | **November** | **December** |
| --- | --- | --- | --- | --- | --- | --- | --- | --- | --- | --- | --- | --- |
| **Max Temp (°C)** | **19** | **21** | **25** | **30** | **35** | **38** | **40** | **40** | **36** | **31** | **25** | **20** |
| **Min Temp (°C)** | **7** | **8** | **11** | **15** | **20** | **23** | **25** | **25** | **22** | **18** | **12** | **8** |
| **Avg Temp (°C)** | **13** | **15** | **18** | **23** | **28** | **31** | **33** | **33** | **29** | **25** | **19** | **14** |
| **Humidity (%)** | **62** | **57** | **52** | **42** | **32** | **28** | **28** | **28** | **33** | **42** | **52** | **62** |
| **Rainfall (mm)** | **1** | **1** | **0** | **0** | **0** | **0** | **0** | **0** | **0** | **1** | **1** | **1** |

**2024 — Monthly Climate Averages**

| **Parameter** | **January** | **February** | **March** | **April** | **May** | **June** | **July** | **August** | **September** | **October** | **November** | **December** |
| --- | --- | --- | --- | --- | --- | --- | --- | --- | --- | --- | --- | --- |
| **Max Temp (°C)** | **20** | **22** | **26** | **31** | **36** | **39** | **41** | **41** | **37** | **32** | **26** | **21** |
| **Min Temp (°C)** | **8** | **9** | **12** | **16** | **21** | **24** | **26** | **26** | **23** | **19** | **13** | **9** |
| **Avg Temp (°C)** | **14** | **16** | **19** | **24** | **29** | **32** | **34** | **34** | **30** | **26** | **20** | **15** |
| **Humidity (%)** | **63** | **58** | **53** | **43** | **33** | **29** | **29** | **29** | **34** | **43** | **53** | **63** |
| **Rainfall (mm)** | **1** | **1** | **0** | **0** | **0** | **0** | **0** | **0** | **0** | **1** | **1** | **1** |

Estimated using climatological data from Egypt Tours Portal and World Bank Climate Knowledge Portal. Egypt Tours Portal. (2022 and 2024).
