# Supplementary material for: NPK nano-fertilizers enhance growth, oil quality, and yield regularity in Picual olive trees
Source: Sci Rep. 2025 Sep 12;15:32495. doi: 10.1038/s41598-025-17267-9 (PMC12432146; doi:10.1038/s41598-025-17267-9)
Supplement: Supplementary file 3 — Supplementary Material 3 [file 41598_2025_17267_MOESM3_ESM.docx]

**Supplementary Figure S1
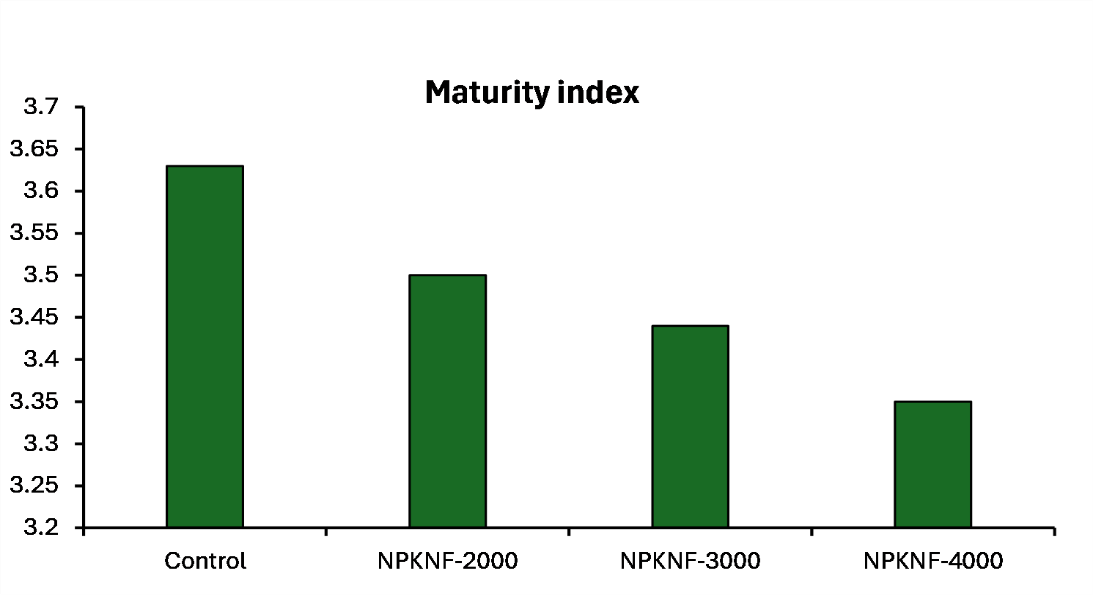
**

**Supplementary Figure S1.** Effect of spraying NPK nano-fertilizers on maturity index of ‘Picual’ olive trees during the "on-year" season.
